# Herpesvirus Infection Induces both Specific and Heterologous Antiviral Antibodies in Carp

**DOI:** 10.3389/fimmu.2018.00039

**Published:** 2018-01-24

**Authors:** Julio M. Coll

**Affiliations:** ^1^Departamento Biotecnología, Instituto Nacional Investigaciones y Tecnologías Agrarias y Alimentarias (INIA), Madrid, Spain

**Keywords:** heterologous, specific, carp, IgM, enzyme-linked immunosorbent assay, viral hemorrhagic septicemia virus, cyprinid herpes virus 3

## Abstract

IgM antibody diversity induced by viral infection in teleost fish sera remains largely unexplored despite several studies performed on their transcript counterparts in lymphoid organs. Here, IgM binding to microarrays containing ~20,000 human proteins was used to study sera from carp (*Cyprinus carpio*) populations having high titers of viral neutralization *in vitro* after surviving an experimental infection with cyprinid herpes virus 3 (CyHV-3). The range of diversity of the induced antibodies was unexpectedly high, showing CyHV-3 infection-dependent, non-specific IgM-binding activity of a ~20-fold wider variety than that found in sera from healthy carp (natural antibodies) with no anti-CyHV-3 neutralization titers. An inverse correlation between the IgM-binding levels in healthy versus infection-survivor/healthy ratios suggests that an infection-dependent feed back-like mechanism may control such clonal expansion. Surprisingly, among the infection-expanded levels, not only specific anti-frgII_CyHV-3_ and anti-CyHV-3 IgM-binding antibodies but also antibodies recognizing recombinant fragment epitopes from heterologous fish rhabdoviruses were detected in infection-survivor carp sera. Some alternative explanations for these findings in lower vertebrates are discussed.

## Introduction

Natural non-specific IgM binding could explain most of the difficulties complicating the estimation of specific antibodies in sera from carp (*Cyprinus carpio*) that survive cyprinid herpes virus 3 (CyHV-3) infection when using indirect enzyme-linked immunosorbent assays (ELISAs) using purified whole virus (1–5). Natural non-specific IgM-binding levels were also reported when using CyHV-3 recombinant proteins for indirect ELISA (6). Furthermore, similar difficulties were found when analyzing sera from fish surviving *novirhabdovirus* infections, such as those caused by viral hemorrhagic septicemia virus (VHSV) or infectious hematopoietic necrosis virus (IHNV) using indirect ELISAs (7–10), including those employing recombinant fragments (11, 12). Such difficulties were generally justified by the “sticky” nature of the IgM molecules to different surfaces and in different fish species (13, 14), despite the addition of background reducing agents (6, 11–13, 15, 16). No characterization of natural (healthy) or infection-dependent non-specific IgM binding has been investigated in fish.

Enzyme-linked immunosorbent assay sera dilutions have proven useful in CyHV-3 serodiagnosis for identifying samples with specific antibodies that range from 300- to 2,500-fold dilution end points. CyHV-3-specific antibodies in infected-survivor sera tend to have relatively high titers of >1,600-fold (4), and titers as high as 62,500-fold have been reported 1 year after natural exposure (2) or as high as 76,800-fold have been reported 8 weeks after experimental infection (17). When sera dilutions of <2,500-fold are used, cross-reactions with CyHV-1 have been observed in some (2, 4) but not all reports (17). Therefore, to best detect infection-dependent non-specific IgM-binding levels, a low dilution of the carp sera was chosen.

Transcriptomic studies have shown that natural IgM repertoires in trout lymphoid organs, as measured by heavy chain antigen-binding CDR3 spectratypes generated by VDJ random combinations (18–20), are characterized by a B-cell polyclonal bell-shaped profile, suggesting the existence of random non-specific natural clones. After VHSV infection, both novel viral-specific dominant clones and new non-specific clones were generated (21, 22). Some of the infection-induced clones were public (common to most fish) whereas others were private (restricted to individual fish) (21, 22). Similar results recently reported for carp infected with CyHV-3 confirmed these data (23). The exploration of IgM-binding levels after viral infection in sera may complement those studies performed at the transcriptomic level in lymphoid organs (21, 23–27) to aid our understanding of how non-specific IgM are generated in fish.

This work focused in the study of both specific and non-specific IgM-binding levels induced by CyHV-3 infection in sera from infection-survivor carp populations having high anti-CyHV-3 neutralization titers. Two main conclusions emerged from these results: (i) natural, non-specific IgM present in healthy sera should be optimally reduced to estimate specific and accurate IgM-binding levels for diagnostic purposes and (ii) fish infection-dependent IgM antibodies and B-cells may generate cross-reactivity properties characteristic of trained immunity, a possibility that has been previously unrecognized even in mammalians. Future work along these lines may help to understand how those complex fish non-specific IgM responses are generated and evolve, and whether or not they may have any importance in the prevention of other diseases.

## Materials and Methods

### Fish Viruses and Cells Used for the Experiments

The CyHV-3 Taiwan strain, isolated at the Graduate Institute of Biotechnology, Central Taiwan University of Science and Technology, Taichung, Taiwan, that affects common and koi carp (*C. carpio*) was kindly provided by Dr. Lee (Taiwan) and used as previously described (6). CyHV-3 was replicated in cells from common carp brain (CCB), kindly provided by the Collection of Cell Lines at the Veterinary Medicine and German Reference Laboratory (Friedrich-Loeffler-Institut, Federal Research Institute for Animal Health, Greifswald-Insel Riems, Germany). The *novirhabdovirus* VHSV-07.71 (28) was replicated in *Epithelioma papulosum cyprini* cells from the fathead minnow *Pimephales promelas* (ATCC, CRL-2872) as previously described (11, 29). Briefly, the abovementioned cell lines were grown at 25°C in a 5% CO_2_ atmosphere with RPMI-Dutch modified cell culture medium that was buffered with 20 mM HEPES and supplemented with 10% fetal calf serum (FCS), 1 mM pyruvate, 2 mM glutamine, 50 µg/ml gentamicin, and 2.5 µg/ml fungizone. To replicate VHSV or CyHV-3, a similar cell culture medium containing 2% FCS was used at 14 or 25°C, respectively. For the *in vivo* infections, supernatants from infected cell monolayers were clarified by centrifugation at 4,000 × *g* for 30 min and stored in aliquots at −70°C. VHSV or CyHV-3 was purified by ultracentrifugation of supernatants from the corresponding infected cell monolayers as previously described (6).

### Source of Sera from Koi Carp (*C. carpio*)

Sera from infected-survivor koi carp were obtained at FLI (Insel Riems, Germany) and kindly provided by Dr. Bergmann. Carp (50–250 g of body weight) were infected by immersion in 10^3^ TCID_50_ of CyHV-3 per milliliters for 2 h (6). All the carp showed clinical symptoms (100% morbidity), and mortalities reached 40% after 1 month. The surviving carp were bled from the tail vein 2 months after infection as previously described (6). To obtain a representative sample of healthy sera from koi carps with no previous history of CyHV-3 infections, serum samples were collected from those carp with no anti-CyHV-3 neutralizing antibodies among those obtained at FLI (Insel Riems, Germany; Dr. Bergmann), Universidad Politecnica (Madrid, Spain; Dr. Torrent), and Cefas Laboratory (Lowestoft, England; Dr. Dixon). Neither VHSV nor CyHV-3 neutralizing antibodies were detected in the carp sera from the different sources.

### Ethical Statement

The fish were handled in accordance with the National and European Guidelines and Regulations on Laboratory Animals Care. Fish work was approved by the corresponding INIAs Ethic Committee (authorization PROEX Oct 2014, 219/14) at INIA aquarium installation number ES280790002069 and handled as provided with permission A/ES/16/I-32. The fish were monitored two to four times daily, and those with external hemorrhages were sacrificed by an overdose of methanesulfonate 3-aminobenzoic acid ethyl ester (MSS2) to minimize suffering. The fish were anesthetized by MSS2 and used for blood extraction. Blood samples were individually collected from the caudal vein, allowed to clot overnight at 4°C, and centrifuged. Supernatant sera were kept frozen at −20°C.

### Determination of IgM Binding of Individual Carp Sera to an Array of 128 Human Proteins

Individual sera from CyHV-3 infection-survivor (*n* = 3) or healthy (*n* = 2) carp were selected among those having high or no titers of anti-CyHV-3 neutralizing antibodies and high or no anti-CyHV-3 IgM antibodies by indirect ELISA against purified CyHV-3 (6). Antibody screening was then performed on the antigen immunology microarray panel of 128 human antigens (composition described on the website of the UTSW microarray core facility) at the UTSW microarray core facility (UT Southwestern Medical Center, Dallas, TX, USA). The human proteins were printed onto 16-pad FAST chip slides. Each chip contains 16 identical arrays for processing 15 samples and 1 phosphate-buffered saline (PBS) control. Solid phases were blocked for 60 min with dilution buffer (0.5% bovine serum albumin, 0.1% Tween-20, 0.01% merthiolate, and 0.005% phenol red in PBS pH 6.7) containing 100 µg/ml (0.01%) of skimmed milk (Sigma). Individual carp serum samples were 100-fold diluted in dilution buffer and incubated with the solid-phase array. The IgM-binding levels were detected with Cy3-labeled anti-carp IgM monoclonal antibody MAb8 (Aquatic Diagnostics, Stirling, Scotland, UK). The arrays were then scanned with a GenePix^®^ 4400A Microarray Scanner and the images were analyzed using GenePix 7.0 software. The averaged fluorescent intensity of each protein spot was normalized to internal controls as described by the manufacturer.

### Determination of IgM-Binding of Carp Sera to an Array of 20,234 Human Recombinant Proteins

Antibody screening was performed on HuProt™ v3—Human Proteome Microarrays (Cambridge Protein Arrays, UK). The HuProt™ v3 Human Proteome Microarray provides yeast *S. cerevisiae* expression products of 20,234 clones encoding human recombinant proteins in duplicate. Carp sera were pooled from either CyHV-3 infection-survivors (*n* = 6) or healthy (*n* = 6) carp because of the high costs of these microarrays. According to the manufacture, the microarray covers ~75% of the annotated human genome and supplies a large array of different random epitopes. Human proteins are expressed as N-terminal GST-RGS-His6 fusion proteins that were purified and printed onto 75 mm × 25 mm SuperEpoxy 2 glass slides. The solid phases were blocked for 60 min with dilution buffer containing a limited amount of skimmed milk as described earlier. Sera pools were 100-fold diluted in the dilution buffer mentioned earlier, to a final assay volume of 3 ml and then incubated with the microarray. Secondary reagents were anti-mouse-IgG-546 and streptavidin-647 conjugated to fluorophores with excitation maxima at 546 and 647 nm, respectively. Anti-GST staining acted as a positive control and provided data on the relative amount of every protein on the microarray. As a negative control to estimate backgrounds, one additional microarray was treated identically to the experimental microarray but was incubated with only dilution buffer instead of a serum sample. Upon completion of the assay, the microarrays were scanned with excitation at 543 nm (for detection of IgM binding to the protein) and at 633 nm (for detection of GST-binding to every protein), and the fluorescence data were collected. GenePix software was used to align the human proteins spotted *via* microarray positioning. For each protein on the microarray, the fluorescence signals that were obtained from the corresponding protein on the negative control microarray that was incubated with reagents, but not with carp sera, were subtracted from the fluorescence signals that were obtained with the sera, yielding corrected protein-specific fluorescence signals. Following these corrections, mean fluorescence signals were calculated from the duplicate spots, values were log2 transformed, and signal significance was determined by following the manufacturer’s instructions (*z*-scores and interaction scores). The values and thresholds used for filtering were the relative SD of duplicate spots, which was <0.35, and the signal-to-noise-ratio of the sample signal which, was >2.5, to be considered significant.

### Design, Construction, and Purification of Recombinant frg11_VHSV_ and frgII_CyHV-3_

The design, construction, expression, and purification of fragment frg11 (amino acid residues 56–110) of the glycoprotein G of VHSV-07.71 strain (AJ233396) were performed as previously described (11, 30). The sequence of the CyHV-3-U reference strain (NC009127) was used as the source of the ORF149 cDNA sequence to design fragment II (amino acid residues 42–159) as described before (6). The fragments frg11_VHSV_ and frgII_CyHV-3_ were selected because of their immunodominance among the VHSV (11, 12) and CyHV-3 (6) epitopes, respectively. Alignment of the 119 amino acids of frgII_CyHV-3_ and the 56 amino acids of frg11_VHSV_ resulted in only a 12% match (15 conserved positions randomly distributed throughout both sequences) (Clone Manager vs9, Denver, CO, USA), minimizing possible cross-reactivities among them. Recombinant frg11_IHNV_ and frg11_SVCV_ were similarly obtained from homologous frg11_VHSV_ sequences in the corresponding glycoprotein genes within genomes NC001652 and DQ491000, respectively. The nucleotide frg sequences were chemically synthesized (GeneArt, Regensburg, Germany) and subcloned into pRSETa expression vectors that added poly-histidine tag tails to their amino-terminal ends. The plasmids were then transfected into *E. coli* BL21, cultured, and induced with IPTG followed by the purification of extracts by Ni^2+^ affinity chromatography as previously described (6, 11, 30, 31). Purified frgs were kept frozen in 20 mM sodium acetate pH 4.5 to increase their solubility. Gradient polyacrylamide gel electrophoresis in SDS showed >95% purity of the recombinant frgs as estimated by Coomassie blue staining.

### Determination of Total IgM Concentrations in Carp Sera

Concentrations of total IgM were estimated in carp sera by sandwich and/or indirect ELISA. For the sandwich ELISA protocol, plates were coated at 37°C to dryness with 2 µg/well of protein A-affinity purified anti-carp IgM MAb8 (Aquatic Diagnostics Ltd., Stirling, Scotland, UK) (6) diluted in water. Coated solid phases were blocked for 60 min with 100 µl/well of dilution buffer (0.5% bovine serum albumin, 0.1% Tween-20, 0.01% merthiolate, and 0.005% phenol red in PBS pH 6.7) containing 10 µg/well of skimmed milk (Sigma). Sera from healthy and infected fish were 100-fold diluted in dilution buffer, and 50 µl of serum was incubated with the blocked, coated solid phases for 60 min at room temperature. The corresponding rabbit polyclonal anti-IgM labeled with peroxidase (Aquatic Diagnostics, Stirling, Scotland, UK) was used to estimate total IgM binding as will be described in detail below. For the indirect ELISA protocol, plates were coated with 200-fold water-diluted fish sera, and IgM binding was assayed using anti-carp IgM MAb8 and peroxidase-labeled rabbit anti-mouse IgGs (RAM-PO, Sigma Chem. Co., St. Louis, MO, USA) (6). The results of the sandwich and indirect ELISAs were similar (data not shown).

To convert the absorbance values abovementioned assays to IgM concentrations, IgM standard curves were obtained by assaying different concentrations of purified IgM from carp sera. Carp IgM was purified from 10 ml of sera by either affinity chromatography on protein-A-Sepharose columns or precipitation with ammonium sulfate followed by Sephacryl-S300 chromatography. Purity of 80–90% was confirmed by SDS-denaturing gel electrophoresis stained with Coomassie blue and/or Western blotting using the corresponding anti-IgM MAbs (not shown). Serial dilutions of purified IgM created the corresponding standard calibration curves to convert absorbance values at 492–620 nm in micrograms of IgM/ml by Sigmoidal fit (Origin Pro 2016, Northampton, MA, USA) (Figure S1 in Supplementary Material). Standard curves were assayed in the same plate of serum samples or in parallel ELISA using the same buffers and conditions. In addition at least two sera samples were used in all experiments for normalization purposes. Means and SDs were then calculated from three to four independent IgM serum determinations.

### Determination of Heterologous and Specific Antiviral IgM-Binding Levels in Carp Sera

Recombinant frg11_VHSV_ and frgII_CyHV-3_ were used as solid phases to investigate possible heterologous and specific IgM binding, respectively, in sera from CyHV-3 infection-survivor and healthy carp. For comparison purposes, individual sera from CyHV-3 infection-survivor and healthy carp were always analyzed in the same ELISA plate. After testing carp sera at 50-, 100-, and 200-fold dilutions, 100-fold dilution was chosen as the best compromise between infected and healthy sera to discriminate non-specific IgM binding against the recombinant frg11_VHSV_ and frgII_CyHV-3_. The 100-fold diluted carp sera were first preincubated with *E. coli* extracts, a source of abundant CyHV-3-independent epitopes, to reduce background. The concentration of the *E. coli* extract was adjusted so that the sera from healthy carp populations yielded mean absorbance readings at ~0.2 U at 492–620 nm. Unless described otherwise in the figure legends, most of the ELISAs were performed as follows. Individual serum from CyHV-3-infection-survivors and healthy carp were first incubated with solid phases coated with recombinant frg11_VHSV_ from the heterologous VHSV. The resulting supernatants were then incubated with solid phases coated with recombinant frgII_CyHV-3_ from homologous CyHV-3, following a modified ELISA method (6). ELISAs with both sera-incubated frg11_VHSV_ and frgII_CyHV-3_ solid phases were then completed to estimate heterologous and specific IgM binding, respectively (see the scheme in Figure 3).

Polystyrene Maxisorb (Nunc) 96-well plates were coated with 50 µl of water containing 2 µg/well of purified recombinant frg11_VHSV_ or frgII_CyHV-3_ and dried overnight at 37°C. Coated solid phases were first blocked with 100 µl/well of dilution buffer (0.5% bovine serum albumin, 0.1% Tween-20, 0.01% merthiolate, and 0.005% phenol red in PBS pH 6.7) containing 10 µg/well of skimmed milk (Sigma) for 60 min. On the other hand, sera were 100-fold diluted in dilution buffer containing 2 mg of protein/ml of *E. coli* extracts (supernatant from sonicated *E. coli* BL21 centrifuged at 10,000 × *g* for 20 min) and preincubated for 60 min. The amount of *E. coli* extracts was previously determined to reduce the absorbance of the healthy carp sera population to a mean of 0.2 U. Preincubated and -diluted carp sera (50 µl/well) were then incubated for 60 min in frg11_VHSV_ solid phases, and the resulting supernatants were transferred to solid phases coated with frgII_CyHV-3_ and incubated for 60 min. To continue with the ELISA, both sera-treated frg11_VHSV_ and frgII_CyHV-3_ solid phases were incubated with anti-carp IgM MAb8 and peroxidase-labeled rabbit anti-mouse IgGs (RAM-PO, Sigma Chem. Co., St. Louis, MO, USA) for 30 min. After three washes, the color reactions were developed by adding 1 mg/ml *o*-phenylenediamine in citrate buffer containing 3 mM H_2_O_2_. To avoid saturation of the increasing absorbance values with time, the color reactions were stopped after 7 min by adding 3 M H_2_SO_4_. Raw absorbances were measured by using 492–620 nm dual-wave length to correct for individual well differences. Individual raw absorbance values were then normalized to the calculated mean absorbance of the corresponding healthy sera population by the following formula: raw individual absorbance value × 0.2/mean absorbance of *n* healthy sera. To address individual carp variations, sera from populations *n* > 30 carp were tested individually, and each carp population was characterized by a polynomial fitted profile as described before (6). The means and SDs were then calculated from two to four independent determinations. Normalized ELISA absorbance values for each sera fish population were classified in 0.08 absorbance classes, and the resulting relative frequencies were adjusted by polynomial fitting (OriginPro 2015) as described before (6). The differences between two populations were considered significant at *p* < 0.05 (Student’s *t*-test).

### Determination of Anti-CyHV-3 Neutralizing Antibodies in Carp Sera

Common carp brain cell monolayers plated onto poly-d-Lys coated wells (Corning, New York, NY, USA) were used for CyHV-3 micro-neutralization assays by the high-throughput method (32). Briefly, de-complemented (45°C 30 min) fish sera were preincubated with 300 focus forming units of CyHV-3 per well in separate plates overnight. Then, the virus–carp sera mixtures were added to the cell monolayers and incubated for 4 days at 25°C. Next, the infected cell monolayers were fixed with formaldehyde, permeabilized with saponin and stained with a mix of anti-CyHV-3 monoclonal antibodies (6) and fluorescent rabbit anti-mouse IgGs (33). Infected CCB cell suspensions were obtained by limited trypsin digestion of the fixed cell monolayers to be analyzed in a BD FACS Canto II apparatus (Becton Dickinson, San Agustin de Guadalix, Madrid, Spain) provided with a high-throughput sampler. The number of fluorescent cells (CyHV-3-infected cells) over a threshold containing 95% (mean + 2 SDs) of healthy CCB cells was then determined. The percentage of infected cells was calculated using the following formula: 100 × number of cells with fluorescence above the threshold/total number of cells gated per well. CyHV-3-infected cell controls in the absence of any added carp sera showed that 50–70% of the CCB cells were infected, depending on the experiment. The results were then expressed in% of neutralization by the following formula: 100 − 100 × percentage of infected cells/percentage of fluorescence cells in healthy controls. The means and SDs were then calculated from two to four independent determinations.

## Results

### Sera from CyHV-3 Infection-Survivors Showed a Wider Repertoire of IgM-Binding Levels than Those from Healthy Carp

To study the CyHV-3 infection dependence of non-specific IgM binding, eight sera from CyHV-3 infection-survivor carp having the highest CyHV-3 neutralization and anti-frgII_CyHV-3_ titers and eight sera from healthy carp having only natural antibodies (6), were first selected. In the absence of any preincubation of the sera with *E. coli* extracts, their IgM-binding levels were preliminarily explored with a panel of five proteins unrelated to CyHV-3 (chicken ovotransferrin, chicken ovalbumin, human C-reactive protein, human fibrinogen, and keyhole limpet hemocyanine). Strikingly, the results indicated the presence of significant IgM binding to all unrelated proteins tested in sera from both CyHV-3 infection-survivors and healthy carp (data not shown).

**Figure 1:**
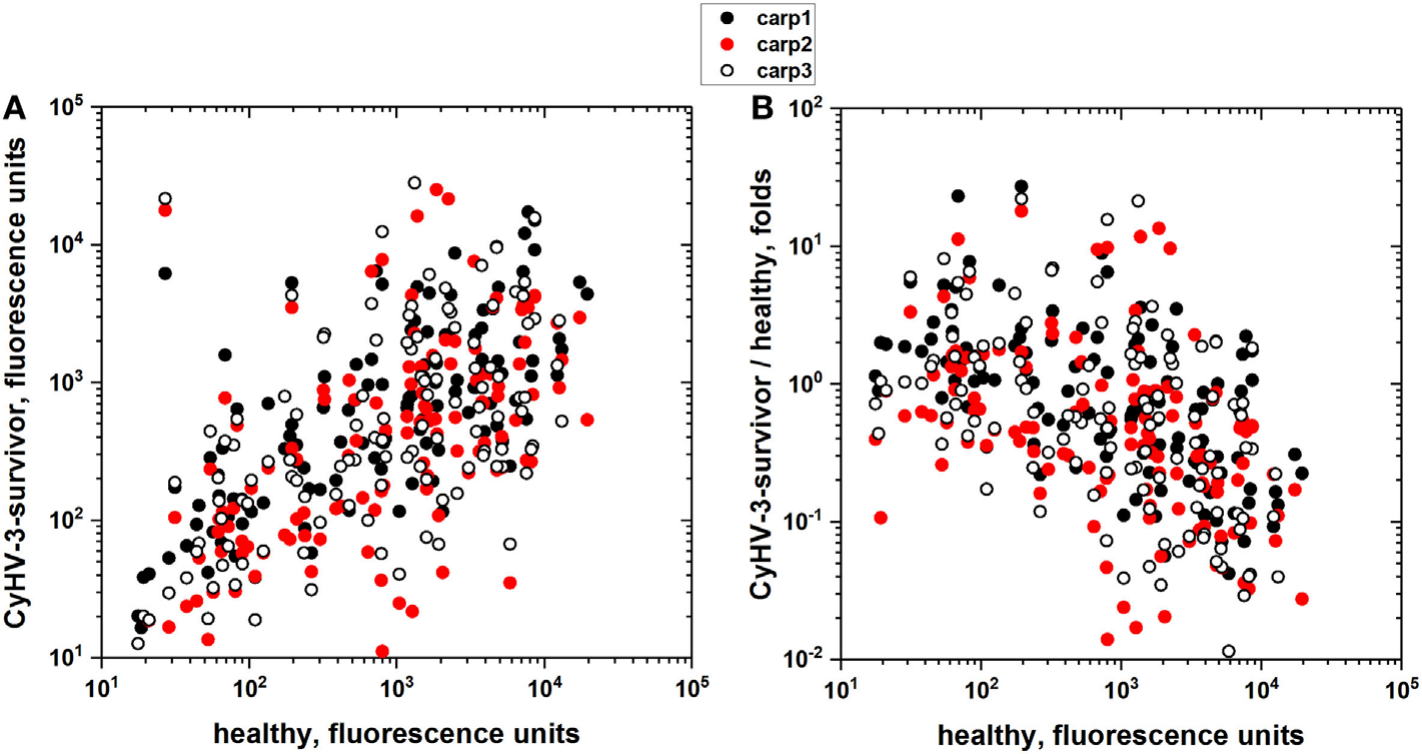
IgM-binding antibodies to an array of recombinant human proteins in the sera from cyprinid herpes virus 3 (CyHV-3) infection-survivor and healthy carp. Individual carp sera from CyHV-3 infection-survivor with high anti-CyHV-3 titers and healthy carp with no anti-CyHV-3 titers were screened for IgM binding to an array of 128 human proteins. Sera from CyHV-3 infection-survivor (*n* = 3) and healthy (*n* = 2) carp were 100-fold diluted and incubated with the protein arrays. IgM binding was detected with the corresponding Cy3-labeled anti-carp IgM MAb8. Backgrounds resulting from arrays incubated in the absence of sera were subtracted. **(A)** IgM binding of sera from CyHV-3 infection survivor versus healthy carp sera. **(B)** Folds versus healthy carp sera, folds calculated for each protein by the formula, fluorescence of CyHV-3 infection-survivor carp sera/mean of fluorescences of the healthy carp sera. Each circle represents one of the proteins in the array. Black circles—IgM binding of CyHV-3 infection-survivor carp 1. Red circles—IgM binding of CyHV-3 infection-survivor carp 2. Open circles—IgM binding of CyHV-3 infection-survivor carp 3.

To explore whether there were more unrelated proteins that were recognized by the carp sera, the IgM binding of 3 CyHV-3 infection-survivor and 3 healthy carp sera were studied using an array of 128 human proteins as a wider source of different epitopes in the absence of *E. coli* extract preincubation. The results showed that each carp serum sample had different distributions of IgM-binding levels for each of the human proteins. However, despite those individual differences, the results also showed a direct correlation between infection-survivor and healthy carp IgM-binding levels (Figure 1A) and an inverse correlation between the IgM-binding levels in infection-survivor/healthy fold ratios versus healthy sera levels (Figure 1B). Taken together, all of the above results suggested that infection-survivor and healthy carp sera contained both CyHV-3-infection-dependent and infection-independent non-specific IgM antibodies, respectively.

**Figure 2:**
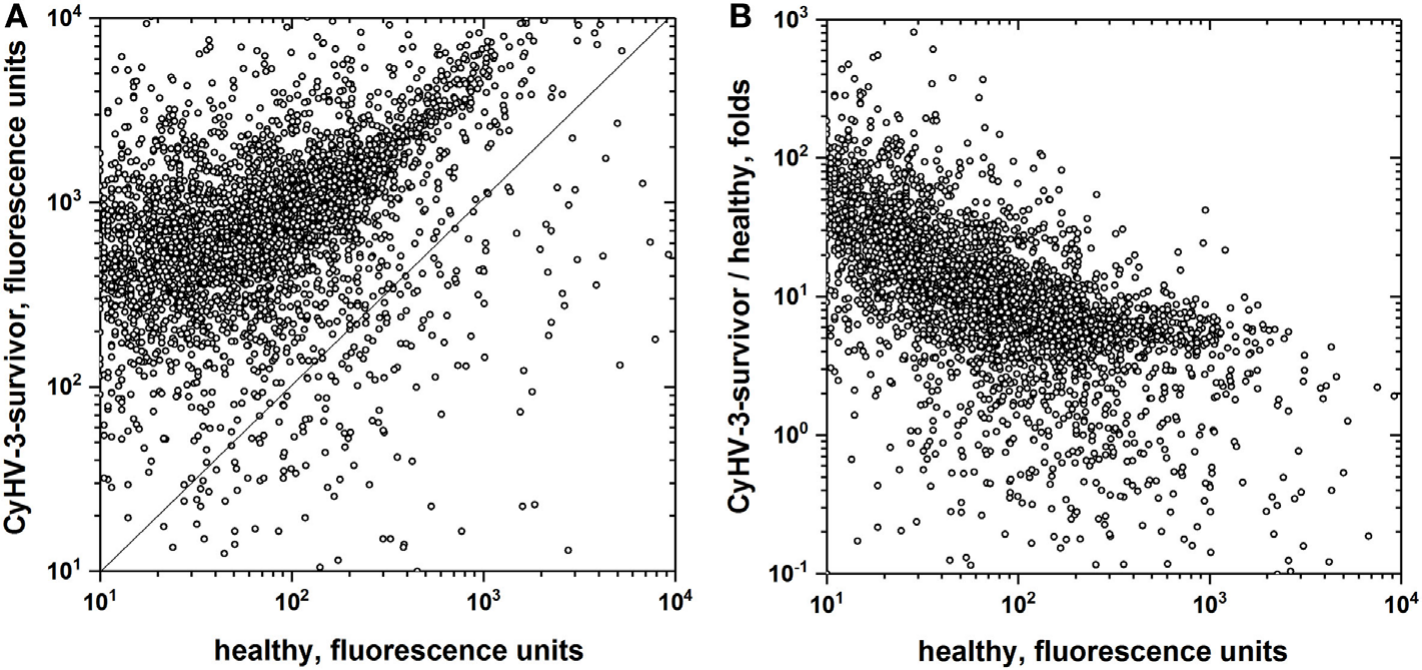
IgM-binding antibodies to a microarray of recombinant human proteins in the sera from cyprinid herpes virus 3 (CyHV-3) infection-survivor and healthy carp. Pooled carp sera from CyHV-3 infection-survivor and healthy carp were screened for IgM binding to a microarray of 20,234 GST-fused recombinant human proteins. Sera were pooled from CyHV-3 infection-survivor (*n* = 6) and healthy (*n* = 6) carp, 100-fold diluted and incubated with the microarrays. Bound IgM was detected with biotinylated anti-mouse-IgG and streptavidin-647. Anti-GST staining was used to normalize the resulting fluorescence raw values. Backgrounds resulting from microarrays incubated in the absence of sera were then subtracted. Mean fluorescences were calculated for duplicate spots, values were log2 transformed and statistical significance estimated. **(A)** IgM binding from CyHV-3 infection-survivor versus healthy carp sera. **(B)** Folds versus healthy carp sera, folds calculated for each protein by the formula, fluorescence of CyHV-3 infection-survivor carp pooled sera/fluorescence of healthy carp pooled sera. Open circles—IgM binding to each of the microarray proteins. Straight line—same CyHV-3 infection-survivor and healthy fluorescence values.

To further explore the extent of non-specific IgM binding in the absence of any *E. coli* extract preincubation, to as many different epitopes as possible, pooled sera from CyHV-3 infection-survivors and healthy carp were bound to the largest available collection of 20,234 recombinant human proteins. The overall results showed 18.6-fold more IgM-binding fluorescence values in CyHV-3 infection-survivor sera than in healthy sera, confirming previous findings reported by other authors for relative antibody levels in VHSV infection-survivor trout (21). While 89.6% of the 20,234 human recombinant proteins were recognized by the IgM from the sera from CyHV-3 infection-survivor carp, only 16.7% of those proteins were recognized by the sera from healthy carp (Figure 2A). While there was a direct trending correlation between CyHV-3 infection-survivor IgM binding and that of healthy sera, an inverse correlation was detected between the IgM-binding levels in infection-survivor/healthy fold ratios versus healthy sera levels (Figure 2B). The former observation confirmed this trend which was supported by the data obtained with the 128 human proteins (Figure 1B), all together suggesting that each of the levels of the CyHV-3 infection-dependent non-specific IgM binding in infected-survivor sera may be controlled by a feed-back-like mechanism that takes into account each of their individual IgM levels before infection.

**Figure 3:**
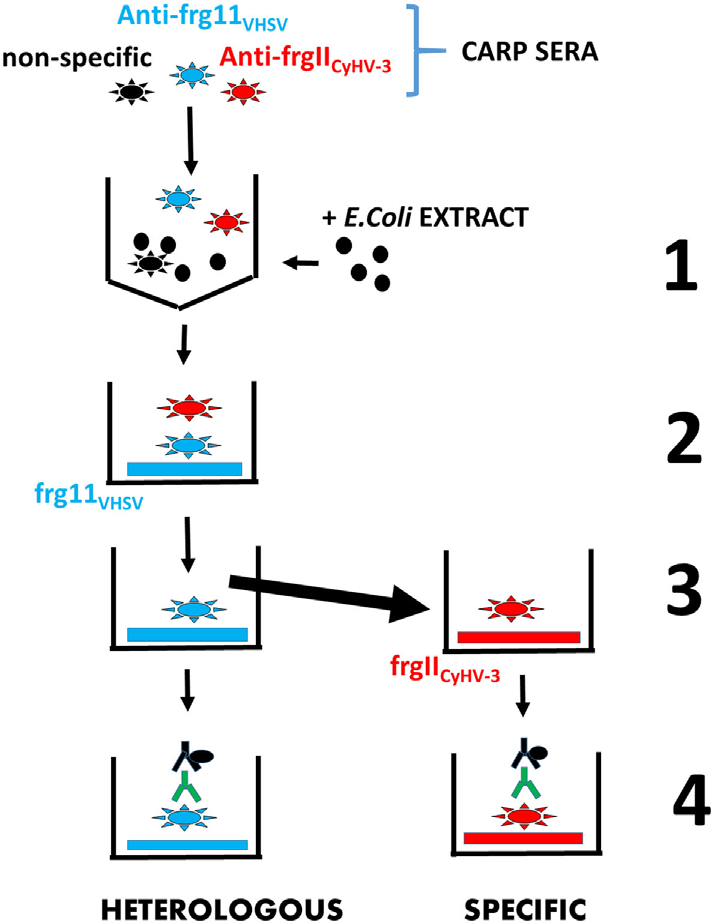
Scheme of the enzyme-linked immunosorbent assay consecutive protocol used to estimate in the same carp sera both heterologous and specific IgM binding to frg11_VHSV_ and frgII_CyHV-3_, respectively. Step 1—carp sera containing IgM against non-specific epitopes (black stars), heterologous recombinant frg11_VHSV_ (blue stars), and specific recombinant frgII_CyHV-3_ (red stars) were blocked with *E. coli* extract (black circles) to reduce mean IgM binding of the healthy carp sera population to a 0.2 absorbance. Step 2—the blocked carp sera were incubated with solid phases coated with heterologous frg11_VHSV_. Step 3—the resulting supernatants were transferred to solid phases coated with specific frgII_CyHV-3_. Step 4—after washing, both heterologous and specific IgM binding were estimated with anti-carp IgM MAb8 and horseradish-labeled rabbit anti-mouse IgG. Black stars—non-specific anti-IgM. Blue stars—heterologous anti-frg11_VHSV_ IgM. Red stars—specific anti-frgII_CyHV-3_ IgM. Black solid circles—*E. coli* extract. Blue horizontal rectangles—recombinant frg11_VHSV_. Red horizontal rectangles—recombinant frgII_CyHV-3_. Green Y—anti-carp IgM MAb8. Black Y + solid circle—horseradish-labeled rabbit anti-mouse IgG.

In view of the expansion of infection-dependent IgM binding to many different heterologous recombinant human proteins, the reactivity of sera populations toward the first panel of purified natural proteins from several unrelated sources was then tested to explore non-specific IgM binding of sera carp populations after preincubation of the sera with *E. coli* extracts to reduce non-specific IgM binding. The raw absorbance results were then normalized, distributed into classes, and polynomically fitted. The results showed that the extent of healthy to infected-survivor shifting of non-specific IgM-binding levels varied with each particular protein tested from no shift (i.e., ovalbumin) to increasing shifting differences (i.e., keyhole limpet hemocyanine to human C-reactive protein) to maximal shift (i.e., human fibrinogen) (Figure S2 in Supplementary Material). These results confirmed that CyHV-3 infection causes an expansion of the natural IgM-binding levels at the population level toward epitopes not present during the infection at variable mean values.

Taken together, all of those results suggested the possibility that sera from CyHV-3 infection-survivors may contain infection-dependent IgM binding not only to homologous viral fragments (i.e., frgII_CyHV-3_) but also to heterologous viral fragments (pathogen cross-reactive antibodies). To further explore the possible existence of such heterologous IgM binding, reactivities of carp sera populations were assayed after preincubation with *E. coli* extracts by using fragments from heterologous fish rhabdoviruses, and the results were analyzed as population profiles.

### Sera from Carp Surviving CyHV-3 Infection Contain IgM That Binds to Heterologous Unrelated frg11_VHSV_

**Figure 4:**
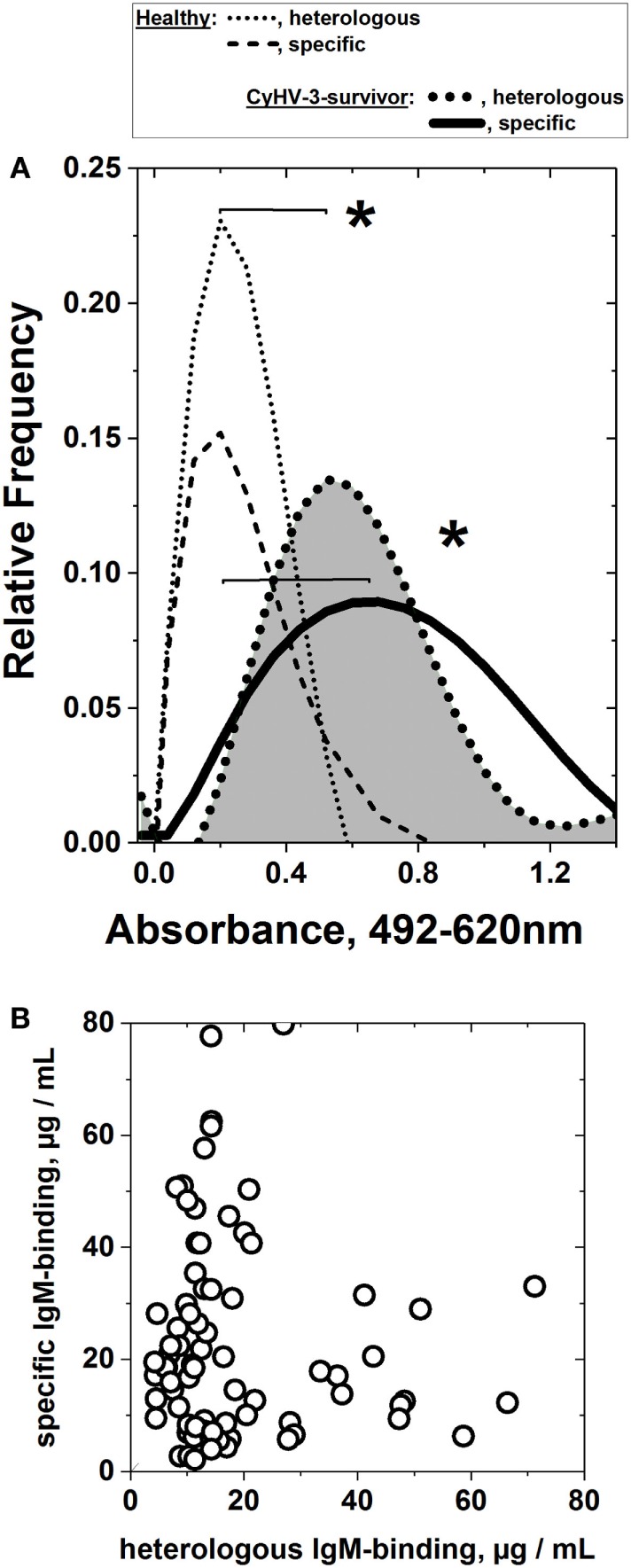
Heterologous and specific IgM binding of sera from cyprinid herpes virus 3 (CyHV-3) infection-survivor carp populations to consecutive frg11_VHSV_ and frgII_CyHV-3_. Individual serum was obtained from CyHV-3 infection-survivors and healthy carp populations, diluted 100-fold and assayed in the same plate by consecutive frg11_VHSV_ + frgII_CyHV-3_ enzyme-linked immunosorbent assay (scheme in Figure 3). Two micrograms of recombinant frg11_VHSV_ or frgII_CyHV-3_ were used as solid phases. Heterologous and specific IgM binding of sera from carp surviving infection with CyHV-3 was estimated after preincubation of the sera with *E. coli* extract at an optimal concentration to lower to ~0.2 absorbance units the mean IgM binding of sera from the healthy carp population. Each raw absorbance value was normalized by the formula, raw individual absorbance value × 0.2/mean of healthy sera (*n* = 65). The IgM binding assays were repeated two to three times and their individual means used for further calculations. After normalization, the values were distributed in 0.08 absorbance classes and polynomically fitted, as indicated in Section “Materials and Methods.” *Statistically significant differences at the *p* < 0.05 level (Student’s *t*-test). **(A)** Heterologous and specific IgM binding of sera from the CyHV-3 infection-survivor carp population. Dotted black line (gray surface)—heterologous IgM binding of sera from the CyHV-3 infection-survivor carp population to frg11_VHSV_ (*n* = 91). Black line—specific IgM binding of sera from the CyHV-3 infection-survivor carp population to frgII_CyHV-3_ (*n* = 91). Black dotted and dashed lines—heterologous and specific IgM binding from the healthy carp sera population, respectively, normalized to 0.2 absorbance means (*n* = 65). **(B)** Lack of correlation between heterologous (*X*-axis) and specific (*Y*-axis) IgM-binding concentrations from the individual sera from the CyHV-3 infection-survivor carp.

Two months after the carp population survived CyHV-3 infection, the sera IgM-binding profiles from the carp population to frg11_VHSV_ and frgII_CyHV-3_ were estimated by ELISA after preincubation of the sera with *E. coli* extracts. Surprisingly the results showed an heterologous IgM binding mean to frg11_VHSV_ similar to that of the CyHV-3 and significantly higher than any of those obtained from sera from healthy carp (compare to healthy carp from CyHV-3 infected-survivor carp data in Figure 4A). It is unlikely that the observed heterologous IgM-binding profile was due to residual *E. coli* present in the purified viral fragment preparations, since both CyHV-3 and healthy sera were preincubated with *E. coli* extracts before ELISA to reduce such possible reactivities, and since the total protein contaminants of the recombinant proteins as estimated by gel electrophoresis after similar purification procedures from *E. coli* transformed with empty plasmids were <5% of the total protein (data not shown). Furthermore, an additional excess of *E. coli* extracts or synthetic polyH tails in the buffers did not reduce the levels of CyHV-3 infection-dependent IgM binding (data not shown). The observed heterologous IgM-binding profile was not related to any previous infections either, since neither VHSV nor CyHV-3 neutralizing antibodies were detected in the carp sera from the different sources. On the other hand, carp was not susceptible to VHSV even when infected experimentally at 14°C, the optimal temperature for VHSV replication (data not shown).

To study any possible correlation between heterologous and specific IgM binding, their concentration levels in sera (Figure S1 in Supplementary Material) were compared for each individual carp, resulting in a lack of correlation between both samples (Figure 4B).

### Heterologous and Specific IgM Binding Did Not Correlated with Anti-CyHV-3 Neutralization Levels, Nor with Total IgM Sera Concentrations

**Figure 5:**
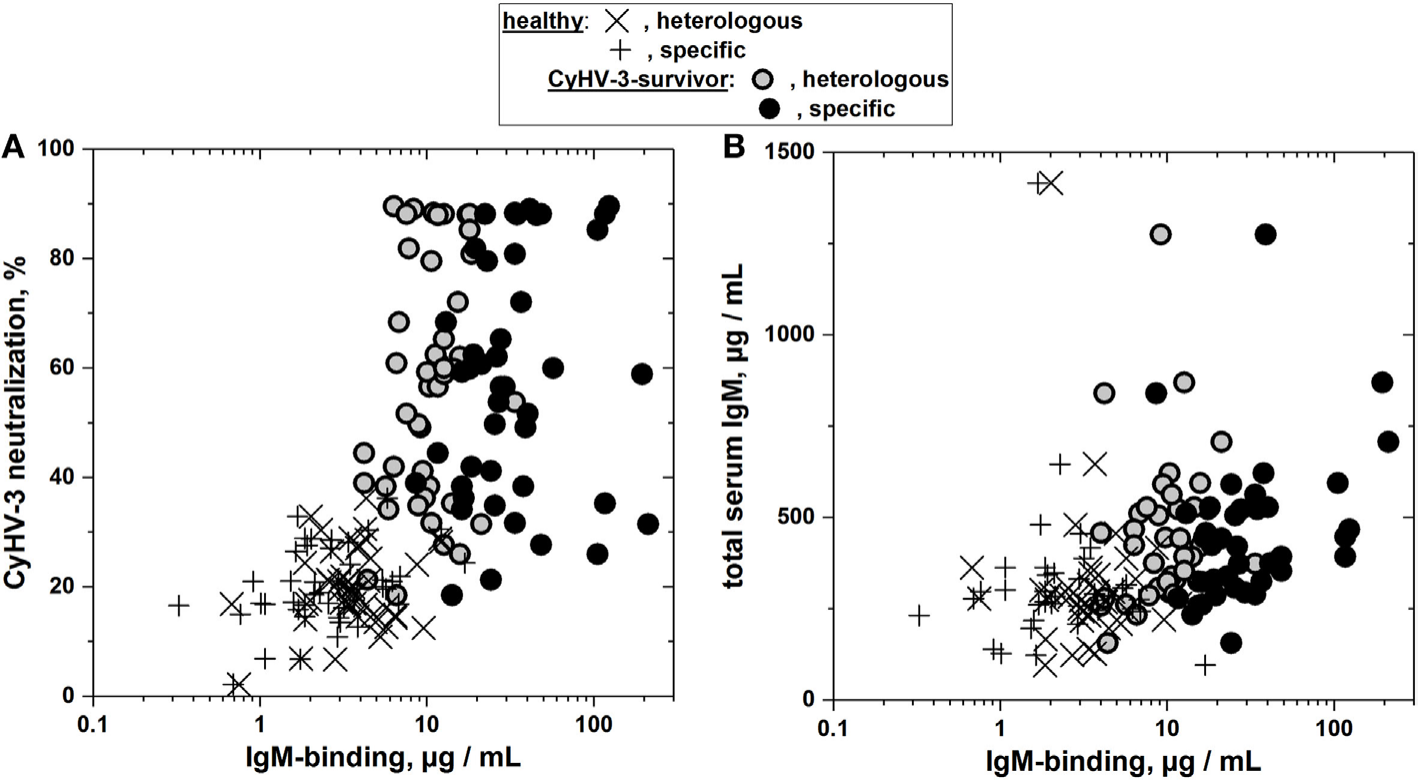
Lack of correlation between heterologous and specific IgM binding and their corresponding neutralization titers **(A)** or total serum IgM levels **(B)** in sera from cyprinid herpes virus 3 (CyHV-3) infection-survivor and healthy carp populations. **(A)** Comparison of heterologous and specific IgM binding with the corresponding percentage of CyHV-3 micro-neutralization. **(B)** Comparison of heterologous and specific IgM binding with the corresponding total concentrations of carp serum IgM. IgM binding of sera from CyHV-3 infection-survivor and healthy carp was determined by consecutive frg11_VHSV_ + frgII_CyHV-3_ enzyme-linked immunosorbent assay. Means from two to four determinations were represented, SDs omitted for clarity. Open circles—gray inside, heterologous IgM binding of sera from CyHV-3 infection-survivor carp (*n* = 43). Black circles—specific IgM binding of sera from CyHV-3 infection-survivor carp (*n* = 43). ×, Heterologous and +, specific IgM binding of sera from healthy carp (*n* = 39 each), respectively.

To explore whether the CyHV-3 infection-dependent IgM binding may correlate to highly specific CyHV-3 *in vitro* neutralizing activities in the carp sera, high-throughput micro-neutralization assays were employed. The relative level of CyHV-3 neutralization and the relative quantity of CyHV-3-specific or non-specific IgM antibodies were not correlated in CyHV-3 infection-survivor carp (Figure 5A), suggesting that these two properties were independently regulated.

To explore whether the CyHV-3 infection-dependent IgM binding may correlate to the concentration of total IgM in the sera, total IgM concentrations were assayed by ELISA (Figure S1 in Supplementary Material). The relative total serum IgM concentration and the relative quantity of CyHV-3-specific or non-specific IgM antibodies were not correlated in CyHV-3 infection-survivor carp (Figure 5B), suggesting that these two properties were independently regulated.

### Heterologous and Specific IgM-Binding Levels Were Also Present against Purified Whole VHSV and CyHV-3 Viruses

**Figure 6:**
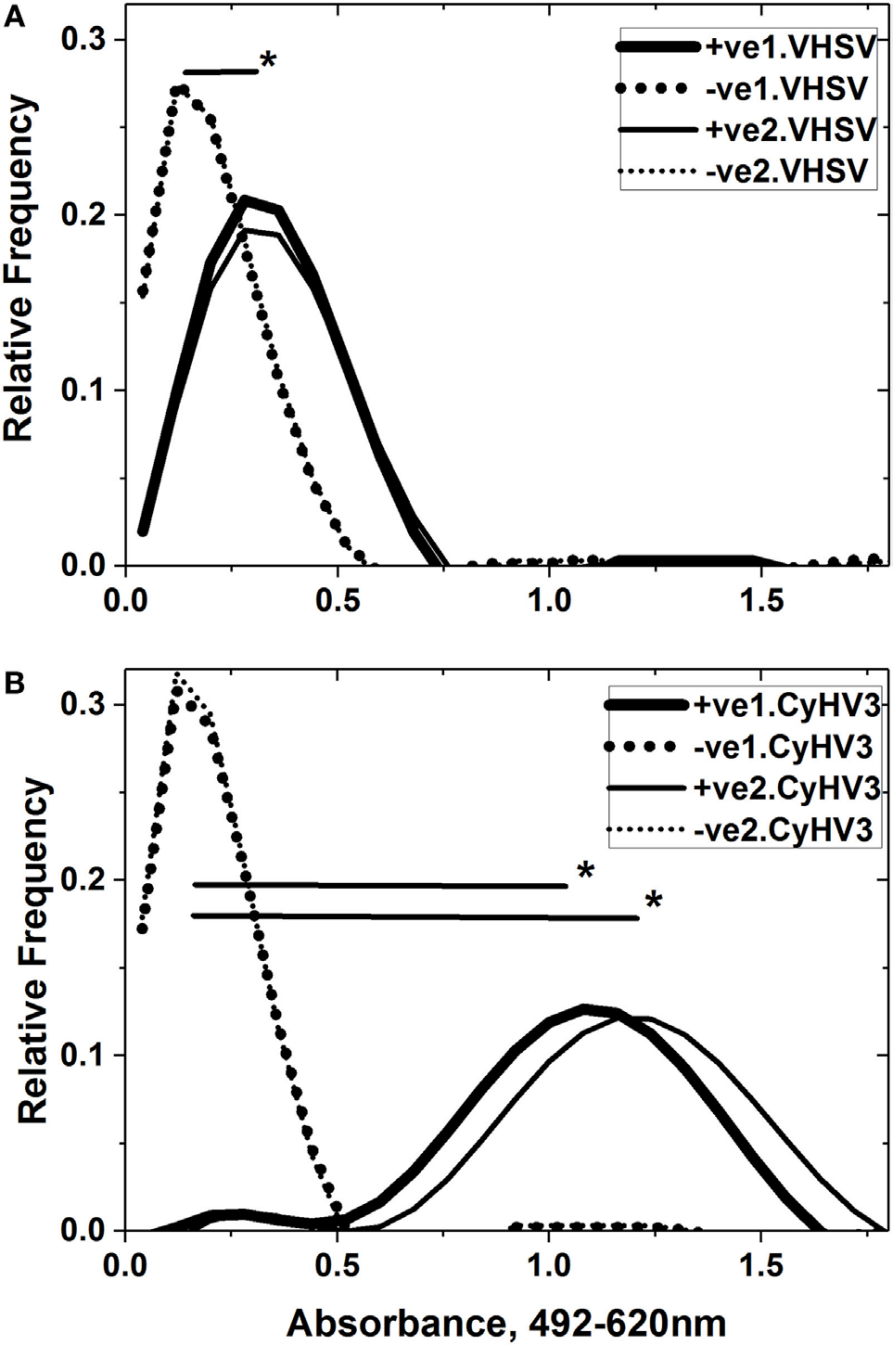
Comparison of consecutive IgM binding to purified viral hemorrhagic septicemia virus (VHSV) **(A)** and cyprinid herpes virus 3 (CyHV-3) **(B)**. Sera from CyHV-3 infection-survivor and healthy carp populations were assayed by consecutive VHSV/CyHV-3 enzyme-linked immunosorbent assay (ELISA) in the same plate. Forward ELISA used VHSV first and CyHV-3 second (wide black lines and dots) while reverse ELISA used CyHV-3 first and VHSV second (thin black lines and dots). Absorbance profiles were obtained after normalization of raw absorbance values, distribution in 0.08 absorbance classes and polynomial fitting, as indicated in Section “Materials and Methods.” Black lines—IgM-binding profiles of sera from the CyHV-3 infection-survivor carp population (*n* = 48). Dot lines—IgM-binding profiles of sera from the healthy carp population (*n* = 48). +ve1.VHSV, CyHV-3 infection-survivor sera population in the first ELISA against VHSV. −ve1.VHSV, healthy sera population in the first ELISA against VHSV. +ve2.VHSV, CyHV-3 infection-survivor sera population in the second ELISA against VHSV. −ve2.VHSV, healthy sera population in the second ELISA against VHSV. +ve1.CyHV3, CyHV-3 infection-survivor sera population in the first ELISA against CyHV-3. −ve1.CyHV3, healthy sera population in the first ELISA against CyHV-3. +ve2.CyHV3, CyHV-3 infection-survivor sera population in the second ELISA against CyHV-3. −ve2.CyHV3, healthy sera population in the second ELISA against CyHV-3. *Significantly >0.2 threshold of the healthy sera population at the 0.05 level (Student’s *t*-test).

To study whether CyHV-3 infected-survivor carp sera populations were capable of recognizing whole VHSV or CyHV-3 and not only their fragments, ELISA was carried out using purified VHSV or CyHV-3 viruses as solid phases. Figure 6 shows that the IgM-binding profiles could be detected against both viruses at significantly higher mean levels than the IgM-binding profiles of healthy sera. The range of IgM binding against CyHV-3 (1.0–1.2 absorbance units) was similar to those reported before (0.9–1.5) at 100-fold dilution of sera from carp surviving other experimental infections of CyHV-3 (2). There were no significant differences regardless of whether the IgM binding was assayed by ELISA first against VHSV (Figure 6A) and then against CyHV-3 (Figure 6B) or *vice versa*. However, the mean absorbance of CyHV-3 IgM binding (Figure 6B) was fourfold to fivefold higher than that against VHSV (Figure 6A) in the CyHV-3 infected-survivor carp population.

### Heterologous and Specific IgM-Binding Levels Were Detected against frg11 from Other Fish Rhabdoviruses

To study whether CyHV-3 infection-survivor carp sera populations were capable of recognizing recombinant frg11 from fish rhabdoviruses other than VHSV, such as IHNV and SVCV, ELISA was carried out using other frg11 from the glycoprotein G of IHNV and SVCV as solid phases. The results showed that IgM binding of CyHV-3 infection-survivor sera recognized all frg11_VHSV_, frg11_IHNV_, and frg11_SVCV_ with differences in their mean levels (Figure S3 in Supplementary Material).

## Discussion

By employing human protein microarrays as an abundant source of different epitopes, this work documents the expansion of both anti-pathogen-specific IgM and non-specific IgM in a polyclonal-like manner at the protein level in carp sera after infection with CyHV-3. The identification of a more limited, but large amount of natural non-specific IgM-binding epitope targets in healthy carp sera was also shown here for the first time. Most likely, natural non-specific IgM binding remained largely undetected up to now because of its elimination by excessive concentrations of blocking reagents in ELISA buffers in serodiagnostic work. The enhancement of the CyHV-3-dependent non-specific IgM-binding expansion compared with healthy carp sera was ~19-fold, similar to the polyclonal transcriptional expansion reported previously in lymphoid organs after infection of trout or carp with VHSV (21) or CyHV-3 (23), respectively. The generation of non-specific IgM binding in fish by a viral infection may be an advantage when being immersed in the pathogen-rich aquatic environment. On the other hand, the lack or lower level of *in vitro* CyHV-3 neutralization observed in some of the CyHV-3 infection-survivor sera in this study and others may reflect an inadequate protection against disease recurrence provided by CyHV-3 specific antibodies in survivor fish (4, 34) and the limited protection observed in carp koi exposed to CyHV-3 following passive immunization with CyHV-3 infection-survivor sera (2). By contrast, the ELISA data in this study and others document an increase in the antibody levels upon exposure of carp to CyHV-3, raising the question of the purpose of this expansion (specific and/or non-specific) if it does not neutralize the virus. Other authors (2) have suggested this may be due to increasing cellular rather than humoral mechanisms of resistance; however, other non-specific protective mechanisms (i.e., trained immunity) could also be involved (see discussion below). This non-specific expansion would explain the existence of false positives on fish serological diagnosis in many reports (6, 11–13, 15, 16) and most of the difficulties experienced with the use of recombinant fragment for serodiagnosis (6), which made the use of whole CyHV-3 the best method to estimate specific carp antibodies for diagnostic purposes (17).

At least part of the natural non-specific IgM binding found in healthy carp populations may be due to the naïve repertoires generated before infection and after VDJ junction random genomic combinations (18, 35, 36), hypermutation (20), and deletion of self-reactive lymphocytes (19). The final non-specific IgM-binding natural antibodies of healthy carp sera before the CyHV-3 experimental infection may have arisen when naïve repertories were modulated by antigen-driven B-cell clonal expansions after individual immunization histories (37).

Analysis of the CyHV-3-dependent IgM protein targets discovered that the lower the level of IgM binding before infection is, the higher the fold increase after infection. This unexpected finding suggested the existence of some epitope-specific feed-back-like regulatory mechanism that controls the relative enhancement of each IgM-producing B-cell clone. A polyclonal-like response could be part of a random, but “preferential” mechanism of expansion for only those B-cell clones that were under represented before infection. Such preferential mechanisms could increase the probabilities of randomly generating pathogen-specific clones in an antigen-independent manner, in addition to the well-known antigen-dependent B-cell expansion (27). This hypothetical preferential mechanism could be more rapid than the antigen-driven expansion or not, but it would generate more possibilities to recognize other epitopes on the infective pathogen and/or in other pathogens.

While confirming the induction by CyHV-3 infection of IgM recognizing specific homologous epitopes (i.e., frgII_CyHV-3_), the exploration at the protein level surprisingly identified heterologous IgM epitope targets, such as those in frg11_VHSV_, and to frg11_IHNV_ or frg11_SVCV_. Because of their higher abundances compared with other non-specific IgM binding, any IgM targeting frgII_CyHV-3_, frg11_VHSV_ or other frgs could be part of the few dominant transcripts of IgM heavy chain clones previously discovered at the transcript level (21, 23). Most likely, the IgM antibodies recognizing the frg11_VHSV_ epitopes were generated randomly among the non-specific IgM polyclonal responses induced by CyHV-3 infection. Alternatively, at least some of the sera from healthy carp may already contain natural antibodies against VHSV and CyHV-3 and/or other viruses or fragments, but most of the corresponding IgM would be of low affinity. CyHV-3 infection may then elicit incomplete maturation of those B-cells to produce more natural antibodies to be detectable only after the expansion induced by infection.

The generation of CyHV-3-dependent anti-frg11_VHSV_ (and any other frgs) IgM could be classified as a pathogen cross-reactivity property. Such cross-reactivity could be generated by several mechanisms. For instance, one possible explanation would be an antigen-independent B-cell polyclonal expansion such as those caused by mitogens, or by T-independent antigens (38–41). Thus, previous examples of polyclonal fish B-cell activation have been described in which salmonid B-cells stimulated by mitogens not only proliferated but also increased total IgM serum levels (42–46). Other alternative explanations may be related to the fact that pathogen cross-reactivity is one of the main characteristics of mammalian trained immunity (47–53). Thus, growing evidence in favor of pathogen cross-reactivity, is now being increasingly identified in mammalians innate responses (50, 51, 53–55) elicited by some vaccines (56, 57). All together those data suggest that it may be possible to develop vaccines of a wide pathogen-spectra (non-specific or heterologous vaccines) (58, 59), rather than pathogen-specific vaccines (57, 59, 60). Mammalian trained immunity cross-reactive responses (50, 51, 53–55, 61), differ from adaptive immune responses in the participation of macrophage and natural killer cells (62–66), rather than adaptive antibody-producing B- and T-cells (62–64). Furthermore, mammalian trained immunity originates by epigenetic reprogramming (DNA/histone modifications, miRNA, etc.), rather than by genetic rearrangements (66, 67). Therefore, the idea of infection-dependent non-specific IgM having some trained immunity-like properties would be a new concept in that it would implicate non-specific B-cells and natural antibodies for the first time. Although, trained immunity-like phenomena in fish remain largely unexplored, a few evidences have been already reported in fish. For instance, DNA vaccines against *novirhabdoviruses* also protected fish against unrelated nodaviruses (68) or rhabdoviruses (69). Furthermore, IgM knockout *rag1^−/−^* mutant zebrafish, maintained memory against secondary bacterial (70, 71) and/or viral (72) infections. On the other hand, β-glucans induced non-specific long-term memory of fish innate immune responses (73), and several non-specific innate immune multigene families were modulated during the rapid memory responses of infected-survivor zebrafish to secondary rhabdoviral infections (74, 75). Could some of the infection-dependent non-specific IgM fish responses be related to new trained immunity-like phenomena? That hypothesis may require the participation of B1-B-cells, in addition to the better known adaptive B2-B-cells (76–81) but there are not yet any supporting evidences, and the abovementioned hypothesis remains speculative. Other explanations apart from those speculated above may also be possible.

Taken together, all of the above commented results suggested that (i) the CyHV-3 infection in carp induces not only specific but also non-specific IgM binding of broader reactivity and (ii) heterologous IgM-binding levels capable of recognizing unrelated whole viruses, such as VHSV, were among the non-specific IgM-binding levels generated by CyHV-3 infection.

## Ethics Statement

The fish were handled in accordance with the National and European Guidelines and Regulations on Laboratory Animals Care. Fish work was approved by the corresponding INIAs Ethic Committee (authorization PROEX Oct 2014, 219/14) at INIA aquarium installation number ES280790002069 and handled as provided with permission A/ES/16/I-32. The fish were monitored two to four times daily, and those with external hemorrhages were sacrificed by an overdose of methanesulfonate 3-aminobenzoic acid ethyl ester (MSS2) to minimize suffering. The fish were anaesthetized by MSS2 and used for blood extraction. Blood samples were individually collected from the caudal vein, allowed to clot overnight at 4°C, and centrifuged. Supernatant sera were kept frozen at −20°C.

## Author Contributions

JC designed, helped in the experimental work, and wrote the manuscript.

## Conflict of Interest Statement

The author declares that the research was conducted in the absence of any commercial or financial relationships that could be construed as a potential conflict of interest.

## Supplementary Material

Figure S1 Conversion of enzyme-linked immunosorbent assay (ELISA) absorbances to IgM concentrations. To convert ELISA absorbances at 492–620 nm from indirect ELISA to IgM concentrations, standard curves were obtained using purified carp IgM as standards. Open circles—carp IgM purified by affinity chromatography over protein-A-Sepharose. Open squares—carp IgM purified by ammonium sulfate and Sephacryl-S300 chromatography. Black lines—sigmoidal fitted curves (Origin Pro 2016).

Figure S2 IgM binding of carp sera populations to several natural proteins. Purified proteins from several natural unrelated sources were obtained from Sigma. Natural proteins were used to coat polystyrene 96-well plates with 2 µg of protein per well. In the same plate, sera from survivor (*n* = 8) and healthy (*n* = 8) carp diluted 100-fold were preincubated with *E. coli* extract and then their corresponding IgM binding estimated. Each raw absorbance value was normalized by the formula, raw individual absorbance value × 0.2/mean of healthy sera (*n* = 8). All normalized values were distributed in 0.08 absorbance classes and polynomically fitted as described in Section “Materials and Methods.” Black solid line—IgM-binding profile of cyprinid herpes virus 3 (CyHV-3) infection-survivor sera population to human fibrinogen. Blue solid line—IgM-binding profile of CyHV-3 infection-survivor sera population to human C-reactive protein. Green solid line—IgM-binding profile of CyHV-3 infection-survivor sera population to keyhole limpet hemocyanine. Light-blue solid line—IgM-binding profile of CyHV-3 infection-survivor sera population to egg ovalbumin. Black dots—IgM-binding profile of healthy sera population (similar profiles for each of the proteins were combined for clarity). *Significantly >0.2 threshold of the healthy sera population at the 0.05 level (Student’s *t*-test).

Figure S3 Comparison of IgM binding to frg11_VHSV_, frg11_IHNV_, and frg11_IHNV_ of carp sera populations surviving cyprinid herpes virus 3 (CyHV-3) infection. Purified recombinant proteins from several fish rhabdoviral frg11 were used to coat polystyrene 96-well plates with 2 µg of protein per well. In the same plate, sera from infected-survivor and healthy carp diluted 100-fold were preincubated with *E. coli* extract and their corresponding IgM-binding estimated. Each raw absorbance value was normalized, distributed in 0.08 absorbance classes and polynomically fitted as described in Section “Materials and Methods.” Black solid line—IgM-binding profile of CyHV-3 infection-survivor sera population to frg11_VHSV_. Blue solid line—IgM-binding profile of CyHV-3 infection-survivor sera population to frg11_SVCV_. Green solid line—IgM-binding profile of CyHV-3 infection-survivor sera population to frg11_IHNV_. Black dots—IgM-binding profile of healthy sera population to frg11_VHSV_. Blue dots—IgM-binding profile of healthy sera population to frg11_SVCV_. Green dots—IgM-binding profile of healthy sera population to frg11_IHNV_. *Significantly >0.2 threshold of the healthy sera population at the 0.05 level (Student’s *t*-test).
